# Bisphenol A and Its Analogues in Chinese Total Diets: Contaminated Levels and Risk Assessment

**DOI:** 10.1155/2020/8822321

**Published:** 2020-12-17

**Authors:** Kai Yao, Jing Zhang, Jie Yin, Yunfeng Zhao, Jianzhong Shen, Haiyang Jiang, Bing Shao

**Affiliations:** ^1^College of Veterinary Medicine, China Agricultural University, Beijing 100193, China; ^2^Beijing Key Laboratory of Diagnostic and Traceability Technologies for Food Poisoning, Beijing Center for Disease Prevention and Control, Beijing 100013, China; ^3^NHC Key Laboratory of Food Safety Risk Assessment, China National Center for Food Safety Risk Assessment, Beijing 100021, China

## Abstract

Bisphenol A (BPA) and its analogues (BPs) are suspected posing potential endocrine disrupting properties. They might migrate into foodstuffs through food packaging materials or contaminated water and soil. Dietary exposure is of paramount importance way for human health. European Food Safety Authority (EFSA) lowered the value of tolerable daily intake (TDI) from 50 *μ*g/kg bw/day (d) to a temporary (t) TDI (t-TDI) of 4 *μ*g/kg bw/d. In this study, the Chinese total dietary samples were analyzed for assessing the exposure risk of BPs by diets. BPA, bisphenol F (BPF), bisphenol S (BPS), and bisphenol AF (BPAF) were found in 12 kinds of food samples except for bisphenol B (BPB). A deterministic approach was used to calculate the dietary intakes of 4 kinds of compounds. For different age and gender groups, the exposure levels of BPA (178.440-403.672 ng/kg bw/d) was the highest, followed by BPS (21.372-52.112 ng/kg bw/d), BPF (20.641-50.507 ng/kg bw/d), and BPAF (0.434-1.210 ng/kg bw/d). Based on the t-TDI set by EFSA (4 *μ*g/kg bw/d for BPA), the BPs through dietary intake pose low risks on the Chinese general population even summarization exposure levels of different BPs. However, human can be exposed to multiple endocrine disrupting chemicals rather than BPs alone; combined exposure risks should be further considered.

## 1. Introduction

Bisphenol A (BPA) is a high-production and high-volume chemical, which is used to manufacture various commodities, such as inner coatings and contact materials of beverage or food due to its rigidity, transparency, and resistance [1]. However, BPA has been shown to be migrated into food, and the migration can be amplified by exposure to alkaline or acidic conditions. Possible sources of human exposure to BPA has been reported, including personal care products [2], teethers [3], environmental water [4], dust [5], air [6], thermal papers [7], drinks [8], and food [9–13]. Diet is the major source of exposure to BPA [9]. An increasing number of studies have shown that exposure to BPA is associated with a variety of toxicities in the neurological [14], endocrine [15], reproductive [16], metabolic [17], and immune systems [18]. Regulations on the production and use of BPA have been brought into force in European Union [19], United States [20], South Korea [21], Japan [22], and China [23]. In 2015, the European Food Safety Authority lowered the value of tolerable daily intake (TDI) from 50 *μ*g/kg bw/day (d) to a temporary (t) TDI (t-TDI) of 4 *μ*g/kg bw/d [19].

Due to the limitation of the BPA use, the bisphenol analogues (BPs), such as BPB, BPS, BPF, and BPAF, were developed as alternatives to BPA and replaced BPA for use in epoxy resins, plastics, thermal papers, and food can linings. As a consequence, BPA analogues were found in several foods (cereals, fruits, meats, etc.) including the commodities from China [24, 25]. However, the endocrine disruptive nature of these analogues seems that they are not less toxic than BPA. Previous reports have shown that the adverse effects of BPs are similar with BPA or more harmful than BPA [26, 27]. Thus, there is increasing demand for the risk assessment of combined exposure of different BPs. Total diet study (TDS) is considered a most efficient and effective method to evaluate the average daily dietary intake of certain chemical substances through the ready-to-eat diet in populations of different ages or genders. The ready-to-eat diet has the advantage of considering loss or introduction of target chemicals and providing analytical results for realistic estimation of dietary intake [28]. In fact, TDS can be used as a priority-setting tool to enable risk managers to focus their limited resources on target compounds. To date, only dietary intakes of BPA in the composite food samples from the Chinese or Canadian TDS were reported [28, 29]. Dietary intake data from TDS on other BPs was not available by far.

The objectives of this work are (i) to investigate the contamination levels of different BPs in the fifth Chines TDS (2009-2013) and (ii) estimate exposure risk among different age and gender Chinese populations.

## 2. Materials and Methods

### 2.1. Reagents and Chemicals

BPA (CAS 80-05-7, purity 98.5%), BPA-*d*_4_ (purity > 97.8%) BPB (CAS 77-40-7, purity > 98.5%), BPF (CAS 620-92-8, purity > 99.0%), BPAF (CAS 1478-61-1, purity 98.0%), and BPS (CAS 80-09-1, purity > 98%) were supplied by Tokyo Chemical Industry Co. Ltd. (Tokyo, Japan). BPF-*d*_10_ (purity > 99%) and BPS-^13^C_12_ (purity > 99%) were purchased from Toronto Research Chemical Inc. (Ontario, Canada), and BPAF-*d*_4_ (purity > 99%) was available from CDN Isotopes Inc. (Quebec, Canada). BPA-*d*_4_ (purity > 99%) and BPB-^13^C_12_ (purity > 99%) were obtained from Cambridge Isotope Laboratories Inc. (Andover, MA). LC-MS grade methanol (MeOH) and acetonitrile (ACN) were offered by Sigma−Aldrich (St. Louis, MO). Ultrapure water was obtained from a Milli-Q ultrapure system (Millipore, Bedford, MA, USA). Phosphate-buffered saline (PBS, 0.2 M) was purchased from Solarbio Science & Technology Co., Ltd. (Beijing, China). The stock standard solutions (10 *μ*g/mL) were individually prepared by dissolving in MeOH and were stored at −20°C. Working solutions were prepared by serial dilution of stock solutions with MeOH/water (50 : 50, *v*/*v*).

### 2.2. Sample Collection

All samples were from the fifth Chinese TDS. The sampling strategy were similar to the fourth Chinese TDS in 2007 [28]. Compared to the fourth Chinese TDS, the sampling sites of the fifth Chinese TDS were enlarged from 12 to 20 provinces (municipalities, autonomous regions), which were geographically divided into four regions. The regions were as follows: North region 1: Heilongjiang (HLJ), Liaoning (LN), Hebei (HB), Jilin (JL) provinces, and Beijing Municipality (BJ); North region 2: Henan (HN), Shanxi (SX), Qinghai (QH) provinces, Ningxia Hui Autonomous Region (NX), and Nei Monggol Autonomous Region (NM); South region 1: Jiangxi (JX), Fujian (FJ), Zhejiang (ZJ) provinces, and Shanghai Municipality (SH); and South region 2: Hubei (HuB), Sichuan (SC), Hunan (HuN), Guangdong (GD) provinces, and Guangxi (GX) Zhuang Autonomous Region. Each region and each province represent a major market basket and a minor market basket, respectively. In each province (municipality, autonomous region), three survey points (two rural and one urban sites, each site has 30 households) was selected. The selected survey points shall be able to represent the general dietary habits, nutritional pattern, and actual dietary structure of residents of each province. The household-based dietary survey adopted a three-day weighed food record method. The consumption foods were classified into 12 groups: cereals and cereal products, legume and related products, potatoes and potato products, meats and meat products, eggs and egg products, aquatic foods and aquatic food products, milk and dairy products, vegetables and vegetable products, fruits and fruit products, sugar and sugar products, beverages and water, and alcohol beverages. Samples of various groups were purchased from markets, grocery stores, shops, and farms near the survey points and were cooked and mixed to form the composites of different food groups similar to the average daily consumption for the population in the province (municipality, autonomous region) according to the results of dietary survey. All samples were stored at -20°C until use.

### 2.3. Sample Preparation

All samples were divided into three broad categories: plant-derived foods (including cereals and cereal products, legume and related products, potatoes and potato products, vegetables and vegetable products, fruits and fruit products, and sugar and sugar products), animal-derived foods (including meats and meat products, eggs and egg products, aquatic foods and aquatic food products, and milk and dairy products), and beverages (including beverages and water and alcohol beverages). The sample preparation methods were based on our previous work [30, 31] with some modifications.

For plant-derived foods, 1 g of homogeneous sample were weighed in 15 mL polypropylene centrifuge tubes. After the addition of ACN (5 mL), the tube was vortexed for 30 s. The sample was then ultrasonically extracted for 20 min and centrifuged at 9184 g for 10 min. The supernatant was transferred to another 15 mL polypropylene centrifuge tube. The extraction step was repeated, and the respective extracts were combined and concentrated to dryness by a gentle stream of N_2_ at 40°C. The residue was redissolved in MeOH/PBS (10 : 90, *v*/*v*; 10 mL), and the solution was vortexed for 30 s. The resultant solution was subjected to IAC cleanup.

For animal-derived foods, 1 g of sample were weighed in 15 mL polypropylene centrifuge tubes. ACN (5 mL) was added. The mixtures were vortexed for 30 s, ultrasonically extracted for 20 min, and centrifuged at 9184 g for 10 min. The supernatants were transferred to fresh 15 mL polypropylene centrifuge tubes; then, PBS (3 mL) was added, and the mixture was stored at −20°C for 3 h. The upper ACN layer was then collected and concentrated to dryness by a gentle stream of N_2_ at 40°C. The residue was redissolved in MeOH/PBS (10 : 90, v/v; 10 mL), and the solution was vortexed for 30 s. The resultant solution was subjected to IAC cleanup.

For beverage samples were firstly degassed in an ultrasonic bath for 30 min. Degassed samples (4 mL) were diluted with 16 mL PBS and adjusted to pH 8.5 with sodium hydroxide solution (0.2 M). The resultant solution was subjected to IAC cleanup.

The prepared solution was loading onto the IAC, then washed with PBS (9 mL) and water (9 mL). Finally, MeOH (3 mL) was used to desorb BPA and BPs. The MeOH-containing analytes were collected and evaporated under nitrogen at 40°C. The residue was redissolved with MeOH/water (30 : 70, *v*/*v*; 1 mL), and the solution was centrifugated at 9000 rpm and transferred 800 *μ*L into vial, then analyzed by ultrahigh-performance liquid chromatography tandem mass spectrometry (UHPLC-MS/MS).

### 2.4. Instrumental Analysis

UHPLC-MS/MS analysis was carried out using a UHPLC system (Nexera X2, Shimadzu, Japan) coupled with a Shimadzu LC-MS 8060 triple quadrupole mass spectrometer (Kyoto, Japan). UHPLC separation was performed on a Waters ACQUITY BEH C18 column (2.1 mm × 100 mm; 1.7 mm; MA, USA). The column temperature was set at 40°C with a flow rate of 0.3 mL/min, and the injection volume was 5 *μ*L. The mobile phases consist of methanol and water. The initial gradient conditions were 30% methanol, followed by a linear increase to 100% methanol in 6 min, and then changed to isocratic conditions with 100% methanol for 2 min. Subsequently, the mobile phase was decreased to 30% methanol in 1 min, held for 3 min before the next injection. The MS/MS detection was operated in the negative ionization mode with multiple reaction monitoring. N_2_ was used as the nebulizing gas at flow rate of 3 L/min; heating gas and drying gas flow rate were 10 L/min and 10 L/min, respectively. Interface temperature, desolvation line temperature, and heat block temperature were 300°C, 250°C, and 400°C, respectively. The optimized MS/MS parameters for the target compounds are given in Table S1.

### 2.5. Quality Assurance and Quality Control (QA/QC)

Isotopic-labeled internal standards (BPS-^13^C_12_, BPF-*d*_10_, BPA-*d*_4_, BPB-^13^C_12_, and BPAF-*d*_4_,) were used to compensate for the matrix effect and recovery loss during the whole analytical procedure. BPS, BPF, BPA, BPB, and BPAF in the food samples were quantified using calibration curves (*r* > 0.999) that were established with seven different concentrations of target standards (0.05-5.00 ng/mL for BPS and BPAF, 0.50-50.0 ng/mL for BPA and BPB, and 1.00-100 ng/mL for BPF). The limits of quantification (LOQs) of BPS and BPAF were 0.05 *μ*g/kg for plant-derived foods and animal-derived foods and 0.013 *μ*g/L for beverages. The LOQs of BPA and BPB were 0.5 *μ*g/kg for plant-derived foods and animal-derived foods and 0.10 *μ*g/L for beverages, respectively. The LOQs of BPF were 1.0 *μ*g/kg for plant-derived foods and animal-derived foods and 0.20 *μ*g/L for beverages, respectively. The recoveries of BPS, BPF, BPA, BPB, and BPAF standards spiked in the matrix sample ranged from 84.6 to 116.8%, 86.4-113.3%, 87.2-116.3%, 87.3-116.7%, and 87.7-117.5%, respectively. The coefficient of variation of all analytes for spiked samples were both below 13.5%.

Blank contamination is a disturbing problem in the ultratrace analysis of BPA and should be avoided in order to achieve low detection limit [32]. Several methods were applied to lower the BPA contamination: (i) glassware were used instead of plastics, and glassware was consecutively rinsed with MeOH and ultrapure water, then baked for four hours at 400°C in a muffle furnace before use (L9/11/B 170, Nabertherm Industrial Furnaces Limited, Lilienthal/Bremen, Germany) [28]; (ii) procedural blanks were analyzed to evaluate background concentration of each analyte, and the concentration was ensured to be below the corresponding LOQ; and (iii) a midpoint calibration standard and methanol were injected every 10 sample injections to check for the instrumental stability, contamination, and the carry-over between samples [33].

### 2.6. Food Consumption and Bodyweight Data

We combined different age (2-7, 8-12, 13-19, 20-50, 51-65, and >65-year-old) and gender groups to estimate daily dietary intakes (EDI) of seven analytes. The data of food consumption (Table S2) and body weights (Table S3) given by the fifth China total diet study [34] and National Health and Family Planning Commission of the People's Republic of China [35, 36] were used to calculate the dietary doses of different food groups.

### 2.7. Estimation of Daily Intake

Estimation of daily intake (EDI) represented the individual dietary exposure of specific age and gender groups of the general population by multiplying the detected levels of target foods and by the average daily food intake of the corresponding food items in each age and gender group [9]. EDI for different age and gender groups was assessed and can be expressed using the following equations:

(1) $$\begin{array}{c} \text{EDI}=\frac{C/w\times \text{FI}}{\text{BW}}, \end{array}$$

where EDI (ng/kg body weight (bw)/day (d)) expresses daily exposure for the different age (2-7, 8-12, 13-19, 20-50, 51-65, and >65-year-old) and gender groups; *C* (*μ*g/kg) expresses the measured mean concentrations of seven analytes in the corresponding food item in the current study, and concentrations below the LOD were replaced by 0.5 LOD, and those below the LOQ but above the LOD by 0.5 LOQ [37]; *w* expresses the water dilution coefficient of the food samples (Table S4); and FI (g/day) expresses the food intake of the corresponding food item by each age and gender group.

## 3. Results and Discussion

### 3.1. EDCs in Foodstuffs

Among 240 composite samples involving in 12 different food items, the highest overall detective rate was found to be 76.7% (BPS), followed by 75.8% (BPA), 29.2% (BPF), 20.8% (BPAF), and 0 (BPB). BPB was not detected in any of the 240 food samples; it is unlikely that BPB have been used in food packaging. This was consistent with previous reports [25, 38]. Furthermore, BPB was also not detected in breast milk and urine samples from Chinese residents [39, 40]. This suggested that Chinese people were not exposed to BPB through dietary or environmental conditions. Concentrations of detected 4 EDCs are listed in Table 1. It is noteworthy that all the samples were cooked and then mixed with water. Therefore, we converted the concentrations of detected EDCs in water-diluted samples into original concentrations of EDCs in uncooked samples based on the water dilution coefficient (Table S4). Although BPA was gradually replaced by its analogues, it was demonstrated that BPA and BPS were the predominant contaminants in foodstuffs. Mean BPA concentrations ranged from 0.815 to 15.073 *μ*g/kg. The BPA concentration of sugar from LN province (69.431 *μ*g/kg) were the highest in all the samples. In China, more than 90% of the sugar production was contributed by sugarcane [41]; the BPA-contaminated sugarcane in LN was extracted and concentrated during the production process leading to the high concentrations of BPA in sugar. Besides, alcoholic beverage samples were all found to be positive; this is due to the migration of BPA from the packaging material (glass bottle or plastic bottle) and the stopper of the glass bottle [42]. Compared to beverages and water, mean concentrations of BPA in alcoholic beverages was higher; it can be interpreted as the higher solubility of BPA in alcohol than in water. Increase levels of BPA was found when comparing with previous Chinese TDS in year 2007 [28]; it can be explained by the fact that growing demand of polycarbonates in consumer goods results in the increase use of BPA. The highest mean concentrations were found in vegetables (mean: 15.073 *μ*g/kg) and fruits (mean: 14.991 *μ*g/kg), followed by sugar (mean: 12.581 *μ*g/kg), which may be due to its widespread presence as environmental contaminants or due to migration from food contact materials. BPA concentrations (1.074 *μ*g/L) in beverages and water in China were consistent with those in Australia [43], French [44], and Portugal [45], but higher than those in Norway [13]. The mean levels of BPA in meat (2.756 *μ*g/kg) and aquatic foods (3.302 *μ*g/kg) are similar to those reported by Zhou et al. [25].

The overall detective rate of BPS was comparable with BPA. The highest BPS concentration (59.915 *μ*g/kg) was detected in legumes and nuts from SX province. The highest mean concentration of BPS was also found in legumes and nuts (4.570 *μ*g/kg), followed by meat (1.774 *μ*g/kg) and aquatic foods (1.635 *μ*g/kg). Compared with nonfat food, concentration of BPS in fat food showed higher levels. The similar trend was found in reports by Zhou et al. [25] and Liao and Kannan [24]. Except for the above three food items, levels of BPS in the rest of food items were significantly higher than those reported by Liao and Kannan [24]. It implies that BPS were increasingly used as a main substitute of BPA in China.

BPF also was detected in various food items (29.2% detective rate). The items of legumes and nuts and eggs contained high concentrations of BPF, and the overall mean concentrations in these two items were 5.248 and 4.040 *μ*g/kg, respectively (Table 1). Mean concentration of BPF in 240 samples ranged from 0.109 to 5.248 *μ*g/kg, which was consistent with those in 289 samples from China [24] and 267 samples from United States [46], respectively. The highest concentration of BPF (94.854 *μ*g/kg) was detected in legumes and nuts from HLJ province. The detective rate of BPAF was the lowest in 4 EDCs. It should be noticed that BPAF was frequently detected in cereals (70.0%) and legumes and nuts (80.0%). There is a conjecture to explain the high detective rates in these food items: crops may absorb and concentrate the BPF and BPAF from their growing environment, such as water, soil, air, and dust [25].

### 3.2. Dietary Intakes of EDCs

The previous studies [9, 44] indicated that dietary intakes of EDCs showed a decrease with the growth of the age. This study shows the same trend in Table 2. The highest and the lowest dietary intakes of each EDCs were in 2- to 7-year-old children and over 65-year-old elders, respectively. We found that there was almost no difference in the dietary intakes of BPA between genders. It should be noted that dietary intakes of people aged 2- to 19 year old (critical time of development) were obviously higher than that of aged above 19. When EDCs are present during development, there is now a growing probability that maternal, fetal, and childhood exposure to chemical pollutants play a larger role in the etiology of many endocrine diseases and disorders of the thyroid, immune, digestive, cardiovascular, reproductive, and metabolic systems [47]. These suggested that EDC exposure has greater impact on pre-teens than in adults [9]. Considering the similar structure and endocrine disrupting properties of BPA and other BPs, the exposure levels of all chemicals were summed up to assess the risks through dietary intake (Table 2); the combined exposure levels (222.337-507.501 ng/kg bw/d) were still below the t-TDI of BPA [19], which indicated the safety of BPA exposure. The exposure levels of BPA (178.440-403.672 ng/kg bw/d) was the highest, followed by BPS (21.372-52.112 ng/kg bw/d), BPF (20.641-50.507 ng/kg bw/d), and BPAF (0.434-1.210 ng/kg bw/d). The order was consistent with those of BPs which was conducted by Zhou et al. [25]. Large amounts of sewage sludge were applied as land every year, and plant-derived food could adsorb EDCs through leaves or roots from the land. Concentrations of EDCs in sewage sludge reported by Song et al. [48] in China were BPA (9.4 ng/g), BPS (4.3 ng/g), BPF (1.9 ng/g), and BPAF (0.4 ng/g); the order was the same as the exposure of EDCs in this study. However, the mean exposure of BPF reported by Liao and Kannan [24] was ca 10 times higher than that of BPS. It can be explained by the fact that the samples collected by Liao and Kannan were from nine cities in China; the dietary characteristics of residents in different cities were not fully considered. The intake of BPA in fifth Chinese TDS in this study (200.955 ng/kg bw/d, mean value for male and female above age of 19) was lower than those reported by Liao and Kannan [24], but higher than the fourth Chinese TDS by Niu et al. [28] and 2008 Canadian TDS by Cao et al. [29]. Compared to the TDS in China and Canada, the increase of BPA exposure in fifth Chinese TDS can be interpreted as rising consumption of BPA. It is reported that BPA consumption in China has increased 10-fold for 2000-2014, to ca. 3 million tonnes per year [49]. Intake of BPA in this study was in the range of those reported by the US National Toxicology Program [50] (8–1500 ng/kg bw/d) and was somewhat lower than those reported by the FAO&WHO [51] (400–1400 ng/kg bw/d) and EFSA [52] (1500 ng/kg bw/d). Difference in food habits may contribute to the high intakes of FAO and WHO and EFSA. Migration of BPA often occurs from epoxy can coatings to contents; canned food is largely consumed as the main part of the diet of people from many countries. It was reported that Chinese ate only 1 kg of canned food per year, but European and American ate 50 and 90 kg per year, respectively [53].

**Figure 1:**
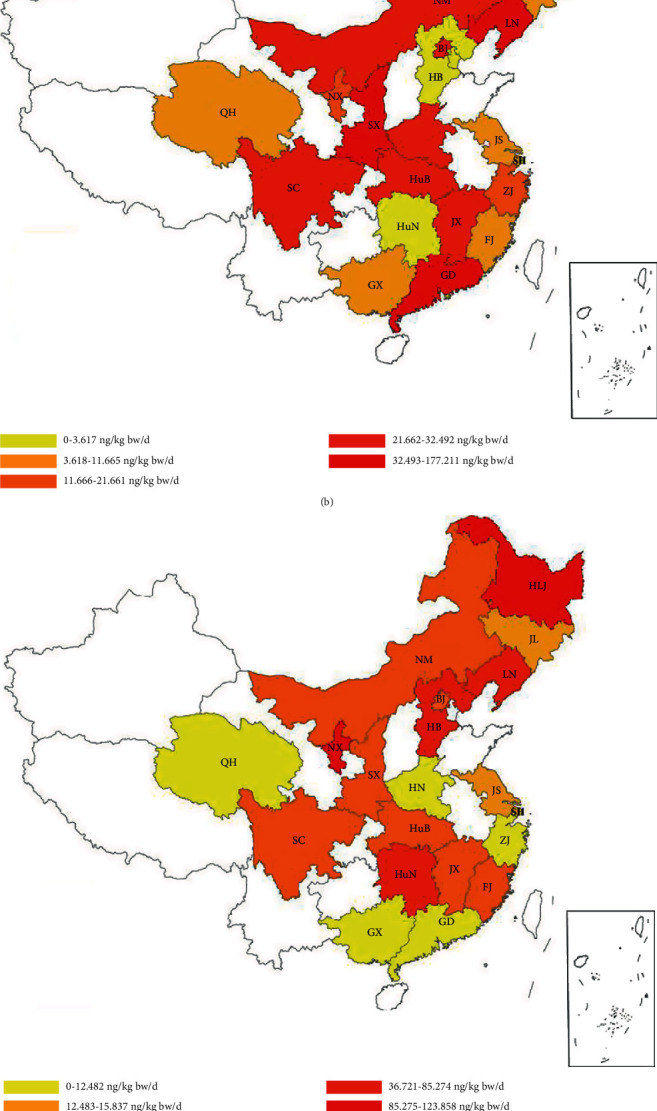
Dietary intakes of (a) BPA, (b) BPS, (c) BPF, and (d) BPAF among 20 provinces.

The EDI of EDCs by the adult residents from 20 provinces were shown in Figure 1. The EDI of BPA was higher among the residents of the industrially developed provinces (JL, LN, JS, ZJ, HuB, SC, and SX) than among those of the other regions. The most likely causes of the above is that higher BPA consumptions of industries in these provinces contributes to the dietary intake. The EDI of BPS was higher in the North 2 and South 2 regions due to the high concentrations of BPS and high consumption of meat in these regions. The EDI of BPF in the north regions was higher than that of the south regions. The high dietary intake may be related to the consumption and concentrations of BPF in legumes and nuts and eggs. The EDI of BPAF among the southeast of China (JL and LN), South I regions (JS, ZJ, and FJ), QH, and HuB shows high dietary intake; this may be explained by the fact that BPAF was used as important chemical raw materials in these industrial provinces.

BPA contributed to the majority of the total intakes of the EDCs, and the mean dietary exposure to other EDCs was as follows: BPS was 25.040 ng/kg bw/d for adult; this was followed by BPF (24.071 ng/kg bw/d), and BPAF (0.505 ng/kg bw/d), respectively. It was higher than those reported earlier (BPS, 1.310 ng/kg bw/d; BPF, 7.460 ng/kg bw/d; and BPAF, 0.275 ng/kg bw/d) [46]. What deserves our attention is the increasing dietary intakes of BPA, BPS, and BPF, while that of BPAF changed less than other EDCs. Because manufacturers have begun to apply BPS and BPF as BPA substitutes in consumer and commercial use, but BPA are still the predominant chemical used in plastics, food packaging, and other products [27].

**Figure 2:**
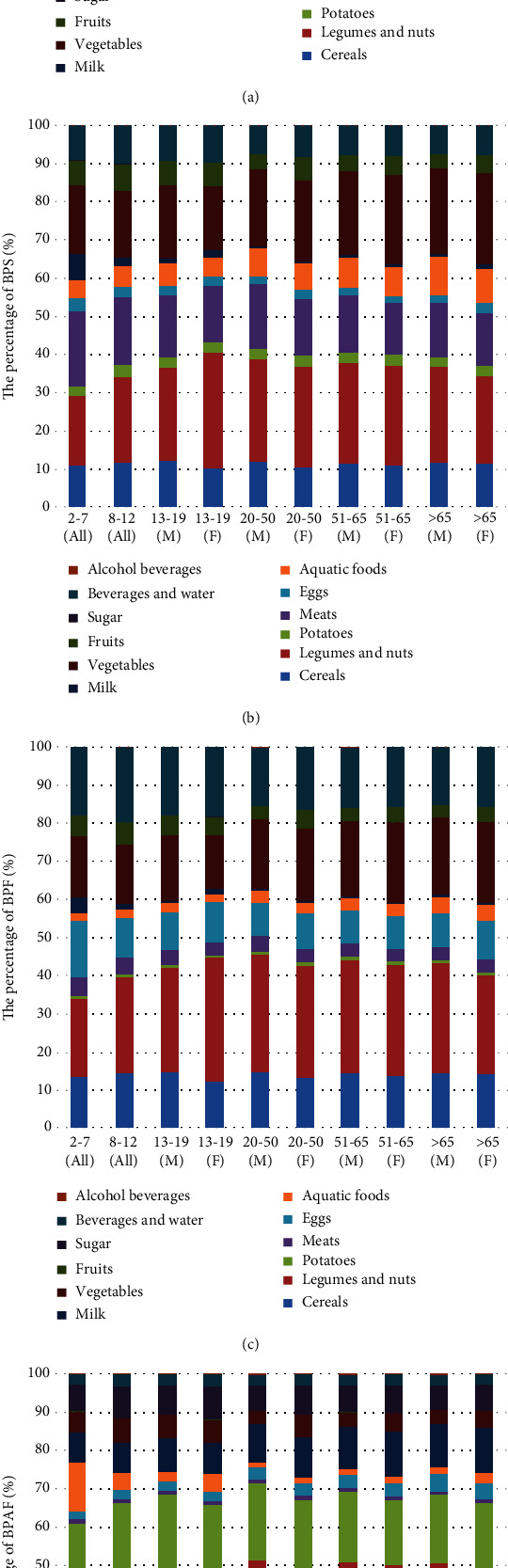
Food items that contribute to total dietary exposure to (a) BPA, (b) BPS, (c) BPF, and (d) BPAF of the different age/gender groups.

The contribution of various food items to total dietary intake of BPA, BPS, BPF, and BPAF are shown in Figure 2 and showed small difference in all age and gender groups. For BPA, the main dietary contributors were vegetables (46.7%-58.4%), cereals (18.1%-21.1%), and fruits (8.9%-17.1%). The results were similar with Niu et al. [28]. For BPS, the main dietary contributors were legumes and nuts (18.4%-30.1%), vegetables (16.5%-23.8%), and meats (13.5%-19.7%). For BPF, the main dietary contributors were legumes and nuts (20.6%-32.3%), beverages and water (15.2%-19.8%), and vegetables (14.0%-21.2%). For BPAF, the main dietary contributors were cereals (33.7%-41.3%) and potatoes (17.1%-21.2%). As shown above, vegetables, cereals, and beverages and water contribute most of the EDC exposure, because these three food items are the major part of people's diet, where the daily consumption are higher than other food. It is important to note that legumes and nuts contribute a lot to the dietary intake of BPS and BPF though its dietary consumption and is not as many as vegetables, cereals, beverages and water. The explanation could be that the BPF is often used in industrial floors and food packaging [27].

## 4. Conclusion

This report surveyed the contamination levels of 5 kinds of EDCs in food samples from the fifth Chinese TDS and found they were detected with varying degrees. Among all the dietary samples, BPB was not found, and detective rates of BPA and BPS were more than 75.8%. The exposure levels of BPA (178.440-403.672 ng/kg bw/d) was the highest, followed by BPS (21.372-52.112 ng/kg bw/d), BPF (20.641-50.507 ng/kg bw/d), and BPAF (0.434-1.210 ng/kg bw/d). This result implied that though BPF has a low detective rate, BPS and BPF were used as main BPA alternatives. The dietary intake of BPA for Chinese people in different age or gender groups was below the TDI. Especially for the children aged 2-7 years old, dietary intakes of all the EDCs were nearly twice the value of people aged above 19 years old, which posed a potential threat to growth and development of children. Coexistence of BPs often happened in food; a mixture of BPs at lower concentration than BPs alone still had estrogen and antiandrogen activity [54]. Though TDI of BPS, BPF, and BPAF have not been set by authorities yet, the health risk caused by the coexposure of BPs should not be overlooked.

## Figures and Tables

**Table 1:** Concentrations of EDCs in different categories of food items collected from 20 provinces in China.

	BPA	BPS	BPF	BPAF
Cereals (*n* = 20)				
Mean (*μ*g/kg)	4.058	0.314	0.352	0.020
Range (*μ*g/kg)	<LOD-14.773	<LOD-2.203	<LOD-1.743	<LOD − <LOQ
Detective rate (%)	75.0	70.0	40.0	70.0
Legumes and nuts (*n* = 20)				
Mean (*μ*g/kg)	0.815	4.570	5.248	0.032
Range (*μ*g/kg)	<LOD-2.908	<LOD-47.932	<LOD-81.034	<LOD-0.188
Detective rate (%)	45.0	65.0	70.0	80.0
Potatoes (*n* = 20)				
Mean (*μ*g/kg)	7.018	0.618	0.218	0.046
Range (*μ*g/kg)	<LOD-42.945	<LOD-7.619	<LOD-1.160	<LOD-0.753
Detective rate (%)	90.0	85.0	10.0	10.0
Meats (*n* = 20)				
Mean (*μ*g/kg)	2.756	1.774	0.416	0.008
Range (*μ*g/kg)	<LOD-12.088	<LOD-10.121	<LOD-1.535	<LOD
Detective rate (%)	80.0	90.0	25.0	0
Eggs (*n* = 20)				
Mean (*μ*g/kg)	5.531	0.747	4.040	0.013
Range (*μ*g/kg)	<LOD-28.720	<LOD-5.339	<LOD-67.831	<LOD-0.100
Detective rate (%)	90.0	90.0	30.0	10.0
Aquatic foods (*n* = 20)				
Mean (*μ*g/kg)	3.302	1.635	0.653	0.016
Range (*μ*g/kg)	<LOD-19.366	<LOD-9.127	<LOD-5.121	<LOD-0.121
Detective rate (%)	70.0	90.0	30.0	10.0
Milk (*n* = 20)				
Mean (*μ*g/kg)	1.107	0.459	0.281	0.008
Range (*μ*g/kg)	<LOD-4.390	0.324-1.217	<LOD-1.710	<LOD
Detective rate (%)	60.0	100.0	20.0	0
Vegetables (*n* = 20)				
Mean (*μ*g/kg)	15.073	0.738	0.621	0.016
Range (*μ*g/kg)	<LOD-29.966	<LOD-2.437	<LOD-2.997	<LOD − <LOQ
Detective rate (%)	70.0	80.0	40.0	45.0
Fruits (*n* = 20)				
Mean (*μ*g/kg)	14.991	0.795	0.641	0.010
Range (*μ*g/kg)	<LOD-26.420	<LOD-2.006	<LOD-8.572	<LOD − <LOQ
Detective rate (%)	80.0	95.0	25.0	10.0
Sugar (*n* = 20)				
Mean (*μ*g/kg)	12.581	0.553	0.280	0.008
Range (*μ*g/kg)	<LOQ-69.431	<LOD-2.633	<LOD-1.602	<LOD
Detective rate (%)	70.0	70.0	10.0	0
Beverages and water (*n* = 20)				
Mean (*μ*g/L)	1.074	0.439	0.852	0.003
Range (*μ*g/L)	<LOD-3.783	<LOD-8.560	<LOD-11.346	<LOD − <LOQ
Detective rate (%)	80.0	30.0	20.0	15.0
Alcoholic beverages (*n* = 20)				
Mean (*μ*g/L)	1.498	0.006	0.109	0.002
Range (*μ*g/L)	0.285-4.487	<LOD-0.029	<LOD-0.982	<LOD
Detective rate (%)	100.0	55.0	30.0	0

**Table 2:** Dietary exposure of EDCs in different gender and age groups (ng/kg bw/d).

	2-7 (all^a^)	8-12 (all)	13-19 (M ^b^)	13-19 (F ^c^)	20-50 (M)	20-50 (F)	51-65 (M)	51-65 (F)	>65 (M)	>65 (F)	Mean (>19, all)
BPA	403.672	331.271	321.082	269.759	199.489	218.320	208.380	221.212	178.440	179.890	200.955
BPS	52.112	42.755	40.622	37.975	26.146	27.365	26.159	26.737	22.463	21.372	25.040
BPF	50.507	41.661	39.707	38.501	24.918	26.478	25.144	25.869	21.374	20.641	24.071
BPAF	1.210	0.936	0.868	0.770	0.539	0.544	0.528	0.529	0.453	0.434	0.505
∑BPs	507.501	416.623	402.279	347.005	251.092	272.707	260.211	274.347	222.730	222.337	250.571

^a^All stands for male and female, ^b^M stands for male; ^c^F stands for female.

## Data Availability

The data used to support the findings of this study are available from the corresponding author upon request.

## Supplementary Materials

Supplementary Materials Table S1: MS/MS parameters of the target compounds. Table S2: food consumption data (g/d) in different age and gender groups. Table S3: bodyweight of people in different age and gender groups. Table S4: water dilution coefficient of the food samples in 20 provinces (municipalities, autonomous regions).
