# Supplementary material for: Bisphenol A and Its Analogues in Chinese Total Diets: Contaminated Levels and Risk Assessment
Source: Oxid Med Cell Longev. 2020 Dec 17;2020:8822321. doi: 10.1155/2020/8822321 (PMC7759395; doi:10.1155/2020/8822321)
Supplement: Supplementary Materials — Table S1: MS/MS parameters of the target compounds. Table S2: food consumption data (g/d) in different age and gender groups. Table S3: bodyweight of people in different age and gender groups. Table S4: water dilution coefficient of the food samples in 20 provinces (municipalities, autonomous regions). [file 8822321.f1.docx]

**Bisphenol A and its analogues in Chinese total diets: contaminated levels and risk assessment**

**Kai Yao^1, 2^, Jing Zhang^2^, Jie Yin^2^, Yunfeng Zhao^3^, Jianzhong Shen^1^, Haiyang Jiang^1*^, Bing Shao^1, 2^***

^1^ College of Veterinary Medicine, China Agricultural University, Beijing 100193, People’s Republic of China

^2^ Beijing Key Laboratory of Diagnostic and Traceability Technologies for Food Poisoning, Beijing Center for Disease Prevention and Control, Beijing 100013, People’s Republic of China

^3^ NHC Key Laboratory of Food Safety Risk Assessment, China National Center for Food Safety Risk Assessment, Beijing 100021, People’s Republic of China

*Corresponding author: [shaobingch@sina.com](mailto:shaobingch@sina.com); or haiyang@cau.edu.cn

Table S1. MS/MS parameters of the target compounds.

| Compound | Precursor (m/z) | Product  (m/z) | Q1 prebar voltage（V） | Collision voltage（V） | Q3 prebar voltage (V) |
| --- | --- | --- | --- | --- | --- |
| BPS | 249.0 | 108.0* | 18.0 | 21.0 | 11.0 |
|  |  | 156.0 | 18.0 | 15.0 | 16.0 |
| BPF | 199.1 | 93.0* | 11.0 | 22.0 | 11.0 |
|  |  | 105.0 | 16.0 | 22.0 | 13.0 |
| BPA | 227.0 | 212.0*  133.0 | 26.0  16.0 | 18.0  25.0 | 25.0  12.0 |
|  |  | 212.2* | 11.0 | 17.0 | 12.0 |
| BPB | 241.2 | 225.1 | 12.0 | 17.0 | 21.0 |
| BPAF | 335.1 | 265.0* | 18.0 | 22.0 | 17.0 |
|  |  | 197.0 | 10.0 | 37.0 | 20.0 |
| BPS-^13^C_12_ | 261.1 | 114.1 | 10.0 | 26.0 | 10.0 |
| BPF-*d*_10_ | 209.2 | 97.1 | 14.0 | 23.0 | 12.0 |
| BPA-*d*_4_ | 231.2 | 216.2 | 12.0 | 19.0 | 14.0 |
| BPB-^13^C_12_ | 253.2 | 223.2 | 10.0 | 29.0 | 14.0 |
| BPAF-*d*_4_ | 339.1 | 269.1 | 12.0 | 23.0 | 17.0 |

* Ion used for quantification.

Table S2. Food consumption data (g/d) in different age- and gender- groups.

| Age (year)/gender group | Cereals | Legumes | Potatoes | Meats | Eggs | Aquatic foods |
| --- | --- | --- | --- | --- | --- | --- |
| 2-7 (All) | 218.3 | 27.6 | 24.3 | 78.7 | 29.6 | 21.8 |
| 8-12 (All) | 336.0 | 48.5 | 44.7 | 102.9 | 30.2 | 37.3 |
| 13-19 (M)^a^ | 461.8 | 70.0 | 49.4 | 125.1 | 37.5 | 54.1 |
| 13-19 (F)^b^ | 368.6 | 80.6 | 48.7 | 104.1 | 39.9 | 41.3 |
| 20-50 (M) | 475.7 | 80.3 | 56.4 | 135.3 | 34.4 | 69.3 |
| 20-50 (F) | 386.9 | 70.9 | 52.9 | 109.0 | 33.4 | 60.1 |
| 51-65 (M) | 461.0 | 78.7 | 55.1 | 120.2 | 33.9 | 74.5 |
| 51-65 (F) | 391.8 | 69.0 | 50.3 | 95.9 | 30.2 | 63.1 |
| >65 (M) | 398.8 | 64.7 | 41.7 | 99.0 | 29.8 | 81.6 |
| >65 (F) | 326.3 | 48.6 | 36.6 | 79.0 | 29.1 | 61.0 |

^a^ “M” stands for “Male”; ^b^ “F” stands for “Female”.

Table S2 Food consumption data in different age- and gender- groups (continued).

| Age (year)/gender group | Diary products | Vegetables | Fruits | Sugar | Beverages and water | Alcohol beverages |
| --- | --- | --- | --- | --- | --- | --- |
| 2-7 (All) | 123.0 | 194.8 | 65.8 | 1.7 | 176.7 | 0.1 |
| 8-12 (All) | 56.0 | 272.4 | 101.0 | 1.3 | 281.3 | 20.9 |
| 13-19 (M) | 39.0 | 396.7 | 122.5 | 0.2 | 340.2 | 5.2 |
| 13-19 (F) | 70.2 | 317.9 | 110.8 | 1.2 | 335.6 | 9.5 |
| 20-50 (M) | 17.8 | 436.4 | 78.0 | 0.3 | 296.6 | 69.1 |
| 20-50 (F) | 22.7 | 412.1 | 107.0 | 0.4 | 296.4 | 8.7 |
| 51-65 (M) | 21.7 | 477.9 | 80.2 | 0.5 | 306.9 | 56.9 |
| 51-65 (F) | 23.9 | 447.0 | 86.2 | 0.4 | 276.3 | 8.3 |
| >65 (M) | 26.1 | 413.3 | 64.4 | 0.1 | 252.5 | 33.0 |
| >65 (F) | 30.2 | 364.1 | 64.7 | 0.2 | 218.8 | 7.0 |

Table S3. Bodyweight of people in different age and gender groups.

| Age (year)/gender group | Bodyweight（kg） |
| --- | --- |
| 2-7 (All) | 16.6 |
| 8-12 (All) | 29.1 |
| 13-19 (M)^a^ | 40.6 |
| 13-19 (F)^b^ | 40.6 |
| 20-50 (M) | 66.2 |
| 20-50 (F) | 57.3 |
| 51-65 (M) | 66.2 |
| 51-65（F） | 57.3 |
| >65（M） | 66.2 |
| >65（F） | 57.3 |

^a^ “M” stands for “Male”; ^b^ “F” stands for “Female”.

Table S4. Water dilution coefficient of the food samples in 20 provinces (municipalities, autonomous regions).

| Food items | Provinces (municipalities, autonomous regions) | | | | | | | | | | | | | | | | | | | |
| --- | --- | --- | --- | --- | --- | --- | --- | --- | --- | --- | --- | --- | --- | --- | --- | --- | --- | --- | --- | --- |
|  | HLJ | HN | HB | BJ | JL | SX | HN | NX | NM | QH | SH | FJ | JX | JS | ZJ | HuB | SC | GX | HuN | GD |
| Cereals | 0.553 | 0.650 | 0.672 | 0.630 | 0.630 | 0.660 | 0.747 | 0.720 | 0.760 | 0.860 | 0.640 | 0.640 | 0.484 | 0.557 | 0.660 | 0.610 | 0.710 | 0.753 | 0.790 | 1.000 |
| Legumes | 0.854 | 0.900 | 0.771 | 0.920 | 0.570 | 0.800 | 0.625 | 0.820 | 0.910 | 0.740 | 0.850 | 0.650 | 0.794 | 0.683 | 0.860 | 0.960 | 0.870 | 0.986 | 0.920 | 1.000 |
| Potatoes | 0.938 | 0.826 | 0.795 | 0.780 | 0.900 | 0.770 | 0.842 | 0.840 | 0.960 | 0.850 | 0.790 | 0.930 | 0.647 | 0.790 | 0.810 | 0.850 | 0.670 | 0.982 | 1.000 | 0.900 |
| Meats | 0.886 | 0.821 | 0.841 | 0.710 | 0.880 | 0.920 | 0.910 | 0.730 | 0.990 | 0.850 | 0.790 | 0.960 | 0.661 | 0.778 | 0.710 | 1.000 | 0.790 | 1.000 | 1.000 | 0.970 |
| Eggs | 0.776 | 0.720 | 0.699 | 0.850 | 0.610 | 0.857 | 0.703 | 0.680 | 0.760 | 0.720 | 0.700 | 0.750 | 0.794 | 0.690 | 0.690 | 1.000 | 0.960 | 0.852 | 1.000 | 0.620 |
| Aquatic foods | 0.954 | 0.914 | 0.792 | 0.870 | 0.970 | 0.880 | 0.670 | 0.950 | 1.000 | 0.890 | 0.700 | 0.820 | 0.704 | 0.894 | 0.920 | 1.000 | 1.000 | 0.976 | 1.000 | 1.000 |
| Diary products | 1.000 | 1.000 | 1.000 | 1.000 | 1.000 | 1.000 | 1.000 | 1.000 | 1.000 | 1.000 | 1.000 | 1.000 | 1.000 | 1.000 | 1.000 | 1.000 | 1.000 | 1.000 | 1.000 | 1.000 |
| Vegetables | 0.993 | 0.912 | 0.982 | 0.980 | 0.820 | 0.890 | 0.943 | 0.940 | 0.970 | 0.810 | 0.910 | 0.920 | 0.938 | 0.795 | 0.910 | 0.930 | 0.810 | 0.958 | 0.996 | 0.990 |
| Fruits | 1.000 | 0.881 | 1.000 | 0.930 | 0.970 | 0.920 | 0.810 | 0.840 | 0.980 | 0.980 | 0.940 | 0.930 | 0.696 | 0.947 | 0.960 | 0.700 | 0.990 | 1.000 | 0.980 | 1.000 |
| Sugar | 1.000 | 1.000 | 1.000 | 1.000 | 1.000 | 1.000 | 1.000 | 1.000 | 1.000 | 1.000 | 1.000 | 1.000 | 1.000 | 1.000 | 1.000 | 1.000 | 1.000 | 1.000 | 1.000 | 1.000 |
| Beverages  and water | 1.000 | 1.000 | 1.000 | 1.000 | 1.000 | 1.000 | 1.000 | 1.000 | 1.000 | 1.000 | 1.000 | 1.000 | 1.000 | 1.000 | 1.000 | 1.000 | 1.000 | 1.000 | 1.000 | 1.000 |
| Alcohol beverages | 1.000 | 1.000 | 1.000 | 1.000 | 1.000 | 1.000 | 1.000 | 1.000 | 1.000 | 1.000 | 1.000 | 1.000 | 1.000 | 1.000 | 1.000 | 1.000 | 1.000 | 1.000 | 1.000 | 1.000 |
